# Insulin resistance in Chinese patients with type 2 diabetes is associated with C-reactive protein independent of abdominal obesity

**DOI:** 10.1186/1475-2840-9-92

**Published:** 2010-12-19

**Authors:** Bin Lu, Yehong Yang, Zhihong Yang, Xiaocheng Feng, Xuanchun Wang, Zhaoyun Zhang, Renming Hu

**Affiliations:** 1 Department of Endocrinology and Metabolism, HuaShan Hospital, Shanghai 200040, PR China; 2 Institute of Endocrinology and Diabetology, Fudan University, Shanghai, PR China

## Abstract

**Background:**

There is debate as to whether the association between C-reactive protein (CRP) and insulin resistance is independent of body fatness, particularly central obesity. Therefore, the association among CRP, insulin resistance and obesity was analyzed in Chinese patients with type 2 diabetes.

**Methods:**

The study included 520 Chinese patients diagnosed with type 2 diabetes with CRP levels not exceeding 10 mg/L. The degree of insulin resistance was determined with the homeostasis model assessment of insulin resistance (HOMA-IR). The CRP levels were categorized into quartiles from the lowest to the highest concentrations (Q1-Q4).

**Results:**

Body mass index (BMI) and waist circumference (WC) were both higher in Q4, Q3 and Q2 than those in Q1. HOMA-IR was higher in Q2, Q3 and Q4 than that in Q1 (Q1 vs Q4, P < 0.001; Q1 vs Q3, P < 0.001; Q1 vs Q2, P = 0.028). Log CRP was significantly correlated with log HOMA-IR (correlation coefficient: 0.230, P < 0.001) and BMI (correlation coefficient: 0.305, P < 0.001) and WC (correlation coefficient: 0.240, P < 0.001) by Spearman correlation analysis. Multiple linear regression analysis adjusting for age, gender and components of metabolic syndrome, log CRP was also independently associated with log HOMA-IR (β coefficient, 0.168; P < 0.001) and WC (β coefficient, 0.131; P = 0.006).

**Conclusion:**

These findings showed that insulin resistance was associated with CRP levels independent of abdominal obesity in Chinese patients with type 2 diabetes, suggesting that abdominal obesity could only partly explain the link between subclinical inflammation and insulin resistance.

## 1. Background

C-reactive protein (CRP), an easily measured marker of systemic inflammation, is stimulated by other cytokines, especially interleukin-6 (IL-6). Insulin resistance is fundamental to the pathogenesis of type 2 diabetes mellitus. During the last few years, insulin resistance has been shown to be strongly associated with CRP and body fat, particularly visceral fat. However, the relationships among obesity, CRP, and insulin resistance are complex. There is debate as to whether the association between CRP and insulin resistance is independent of body fatness, particularly central obesity [1]. Several studies showed that the association of CRP with insulin resistance was independent of obesity [2-6]. Other studies, however, demonstrated that the association between CRP and insulin resistance was obesity dependent in healthy population [7-10]. In these studies, the relationship of CRP and insulin resistance was no longer evident after adjusting for various parameters related to obesity. The aim of our study was to investigate whether insulin resistance is associated with CRP independent of abdominal obesity in the Chinese patients diagnosed with type 2 diabetes.

## 2. Subjects and methods

### 2.1. Subjects

**Figure 1:**
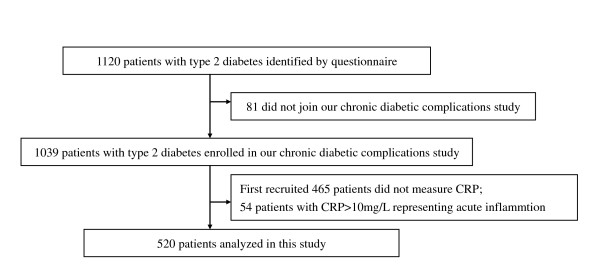
**Screening and Enrollment of Study Subjects**.

Data were obtained from a cross-sectional study undertaken to evaluate the prevalence of diabetic complications in Chinese patients with type 2 diabetes, aged over 30 years and living in downtown Shanghai [11-14]. Twenty residential areas administered by 20 residents' committees were cluster sampled in the central area of Shanghai between 1 February and 31 July 2004. Questionnaires to identify a history of diabetes were sent to every household in these residential areas, and collected by primary care clinicians and endocrinologists. Chinese patients diagnosed with type 2 diabetes were identified by the questionnaire using the diagnostic criteria recommended by the American Diabetic Association in 1997 [15]. Enrolled patients were recruited to the present study and included in the final analysis if their CRP levels had been measured and were ≤10 mg/L (Figure 1). CRP levels >10 mg/L reflect acute inflammation [6], and the present study focused only on patients with low-grade systemic inflammation.

### 2.2. Measurement

Body height and weight were measured from the subjects wearing light clothing without shoes (Horse Head TS120, Shanghai, China). Body mass index (BMI) was calculated from weight in kilogram divided by height in meter square. Waist circumference was measured to the nearest 0.1 cm, using a calibrated plastic tape measure at the umbilical level in the late exhalation phase while standing. The average of the last two measurements of systolic and diastolic blood pressure was obtained from the patients.

### 2.3. Laboratory examination

Using the Hitachi 7600-020 clinical analyzer (Hitachi High-Technologies Corporation, Tokyo, Japan), serum cholesterol was determined by the cholesterol oxidase-paraaminophenazone method (CHO0560, Shanghai Jingyuan Medical Appliances, Shanghai, China), serum triglycerides by the glycerol phosphate oxidase-para-aminophenazone method (TGP0560, Shanghai Jingyuan Medical Appliances), high-density lipoproteincholesterol (HDL-C) by the International Reagents Corporation method (21200AMZ00404000, Daiichi Pure Chemicals, Tokyo, Japan), low-density lipoprotein-cholesterol (LDL-C) by the catalase method (20900AMZ00550000, Daiichi Pure Chemicals), and serum creatinine by the sarcosine oxidase-para-aminophenazone method (S708, Shanghai Kehua Dongling Diagnostic Products, Shanghai, China). Blood urea nitrogen levels were measured using the UVGLDH Method (Cat.No.S507, Shanghai Kehua Dongling Diagnostic Products, Shanghai, China), and uric acid was measured using the uricase-peroxidase coupling method (Cat.No.S710, Shanghai Kehua Dongling Diagnostic Products) according to the manufacturer's instructions.

Glycosylated haemoglobin (HbA1c) was determined by high-pressure liquid chromatography using an automated HLC-723G7 analyser (Tosoh Corporation, Tokyo, Japan). Serum insulin was evaluated by radioimmunoassay, after fasting and then following a 2-h oral glucose tolerance test (Beijing Atom HighTech, Beijing, China). Plasma glucose levels were measured by the glucose oxidase method (GOX0560, Shanghai Jingyuan Medical Appliances) using a Hitachi 7600-020 clinical analyser. Insulin resistance was estimated using the homeostasis model assessment of insulin resistance (HOMA-IR) as follows: fasting serum insulin (mIU/ml) × fasting plasma glucose (mmol/l)/22.5. Serum CRP levels were measured by a chemiluminescence assay using an automatic analyser (Dade Behring, Marburg, Germany). This assay has a sensitivity of 0.162 mg/L. Inter- and intraassay coefficients of variation were <5% for the measurements. For the purposes of this analysis, patients were divided into strata according to quartiles of baseline CRP values(Q1~Q4).

### 2.4. Statistic analysis

Quantitative variables were described as mean ± S.D. One-way ANOVA (multiple comparisons: LSD method) was used for quantitative variables comparisons among the four subgroups according to quartiles of CRP (P < 0.05 considered statistically significant). Chi-square test was used for enumeration data comparisons among the four subgroups according to quartiles of CRP (P value was modified to 0.05/6 based on statistical error of the first kind). The distribution of CRP and HOMA-IR levels were highly skewed. Logarithmically transformed values of CRP and HOMA-IR were used. Spearman correlation analysis was performed to determine the relationship among CRP, HOMA-IR, BMI and waist circumference. To explore the relationship between CRP and HOMA-IR independent of obesity, the association between CRP and HOMA-IR was tested by linear regression analysis with log CRP as the dependent variable. All statistical analyses were conducted with the SPSS statistical package for Windows version 16.0 (SPSS, Chicago, IL).

## 3. Results

A total of 520 patients including 194 male patients and 326 female patients were analyzed in this study. The mean age of these 520 patients was 65.14 ± 12.11 years and the duration of diabetes 7.11 ± 6.73 years.

### 3.1. Clinical characteristics among the four subgroups stratified according to the quartiles of CRP (Q1-Q4)

Table 1 presented the difference of the clinical characteristics among the four subgroups stratified according to the quartiles of CRP (Q1-Q4). BMI and waist circumference were higher in Q4, Q3 and Q2 than these in Q1. BMI was higher in Q3 and Q4 than that in Q2. Systolic blood pressure and diastolic blood pressure were significantly higher in Q4, Q3 than these in Q1 and Q2. Serum cholesterol, triglycerides and LDL-C were higher in Q2 and Q3 than these in Q1. HDL-C was lower in Q2 and Q3 than that in Q1. Gender was not significantly correlated with CRP. HbA1c was also not significantly correlated with CRP. Blood urea nitrogen and serum creatinine were both not significantly correlated with CRP levels. Serum uric acid in Q2 and Q3 was higher than that in Q1 and Q4.

**Table 1:** Clinical characteristics among the four subgroups stratified according to the CRP quartiles

Parameters	Q1	Q2	Q3	Q4
Log CRP (mg/L)	-0.426 ± 0.209	0.062 ± 0.103 *	0.434 ± 0.116 * †	0.781 ± 0.112 * † ‡

CRP (Media)	0.410	1.130	2.850	5.795

Age (years)	62.46 ± 13.46	64.08 ± 12.51	67.25 ± 11.17 * †	66.85 ± 10.56 *

Female/total	78/130	76/130	87/130	85/130

Duration (years)	7.58 ± 7.56	7.22 ± 6.99	6.65 ± 5.76	6.98 ± 6.48

BMI (kg/m2)	23.61 ± 3.14	24.47 ± 3.22 *	25.84 ± 3.22 * †	26.12 ± 3.92 * †

WC (cm)	82.20 ± 8.31	84.85 ± 9.28 *	88.45 ± 8.77 * †	86.92 ± 9.46 *

Systolic BP (mmHg)	130.93 ± 22.64 *	134.73 ± 23.86	140.39 ± 20.62 * †	142.74 ± 20.47 * †

Diastolic BP (mmHg)	77.16 ± 11.12	78.84 ± 10.56	81.84 ± 11.11* †	84.25 ± 10.18 * †

FBG (mg/dl)	144.90 ± 56.52	157.32 ± 63.18	153.00 ± 44.82	154.08 ± 51.48 ‡

PBG(mg/dl)	236.34 ± 100.98	269.10 ± 107.46 *	259.20 ± 93.60	278.10 ± 97.20 *

HbA1c (%)	6.91 ± 1.69	7.40 ± 1.87 *	7.29 ± 1.36	7.23 ± 1.44 ‡

BUN(mg/dl)	17.55 ± 5.45	16.43 ± 4.52	16.76 ± 4.35	17.27 ± 4.89

Cr (mg/dl)	0.89 ± 0.34	0.87 ± 0.22	0.88 ± 0.28	0.88 ± 0.27

UA (mg/dl)	5.01 ± 1.12	5.37 ± 1.37 *	5.54 ± 1.23 *	4.95 ± 1.21 † ‡

TC (mg/dl)	193.69 ± 35.95	209.15 ± 46.78 *	211.08 ± 49.10 *	210.31 ± 47.94*

TG (mg/dl)	138.25 ± 103.25	190.75 ± 168.00 *	196.00 ± 121.63 *	168.00 ± 86.63

LDL-C (mg/dl)	113.66 ± 25.90	124.49 ± 35.95 *	124.49 ± 38.66*	119.46 ± 35.95

HDL-C (mg/dl)	49.48 ± 13.53	45.62 ± 10.44 *	45.62 ± 11.98 *	50.26 ± 14.69 † ‡

Log HOMA-IR	0.457 ± 0.334	0.554 ± 0.355 *	0.636 ± 0.381 *	0.671 ± 0.343 * †

Data were presented as mean ± S.D. except gender and CRP. One-way analysis of variance or Pearson's χ2-test.

CRP: high-sensitivity C-reactive protein; WC: waist circumference; Systolic BP: systolic blood pressure; Diastolic BP: diastolic blood pressure; FBG: fasting blood glucose; PBG: postprandial blood glucose; HbA1c: glycosylated haemoglobin; BUN: blood urea nitrogen; Cr: creatinine; UA: serum uric acid; TC: serum cholesterol; TG: serum triglycerides; LDL-C, low-density lipoprotein-cholesterol; HDL-C, high-density lipoprotein-cholesterol; HOMA-IR, homeostasis model assessment of insulin resistance;

* Significantly different from Q1 subgroups (p < 0.05).

† Significantly different from Q2 subgroups (p < 0.05).

‡ Significantly different from Q3 subgroups (p < 0.05).

### 3.2. Relationships between CRP and HOMA-IR

**Figure 2:**
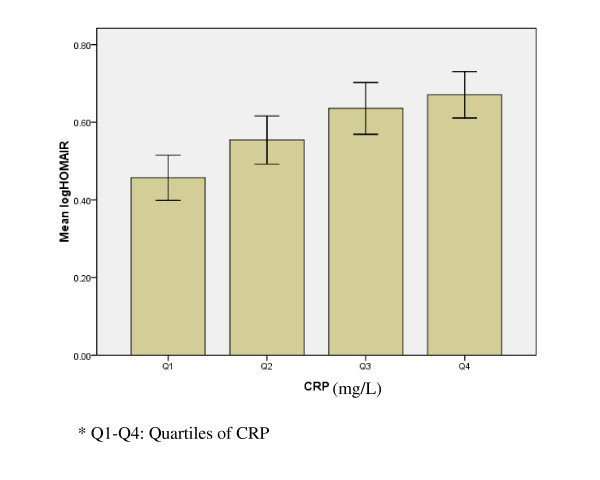
**The distribution of log HOMA-IR index after subjects was classified according to the quartiles of CRP**.

Table 1 also showed that log HOMA-IR was higher in Q2, Q3 and Q4 than that in Q1 (Q1 vs Q4, P < 0.001; Q1 vs Q3, P < 0.001; Q1 vs Q2, P = 0.028). Figure 2 displayed the distribution of log HOMA-IR index after subjects were classified according to the quartiles of CRP.

### 3.3. CRP in relation to HOMA-IR and abdominal obesity

To explore the relationship between CRP and insulin resistance, spearman correlation between log CRP and log HOMA-IR was analyzed. Log CRP was significantly correlated with log HOMA-IR (correlation coefficient: 0.230, P < 0.001). To explore the relationship between CRP and abdominal obesity, spearman correlation between log CRP and BMI, waist circumference was analyzed. Log CRP was significantly correlated with BMI (correlation coefficient: 0.305, P < 0.001) and waist circumference (correlation coefficient: 0.240, P < 0.001).

To explore the relationship between CRP and insulin resistance independent of obesity, the association between CRP and the parameters of insulin resistance was tested by linear regression analysis with log CRP as the dependent variable (Table 2). The following categories including components of metabolic syndrome were taken as independent variables: gender, age, waist circumference, systolic blood pressure, diastolic blood pressure, serum triglycerides, HDL-C, HbA1c and log HOMA-IR. CRP was independently associated with log HOMA-IR (β coefficient, 0.168; P < 0.001) and waist circumference (β coefficient, 0.131; P = 0.006). If waist circumference was replaced by BMI among the above independent variables in the linear regression analysis, CRP was also independently associated with log HOMA-IR (β coefficient, 0.147; P = 0.003) and BMI (β coefficient, 0.181; P < 0.001).

**Table 2:** Multiple linear regression analysis of the relationship between CRP and HOMA-IR

adjusted for the following variables including waist circumference		
	**β coefficient**	**P value**

Gender	0.056	0.207
Age	0.103	0.031
Systolic BP	-0.014	0.823
Diastolic BP	0.187	0.001
Triglycerides	0.054	0.254
HDL-c	0.028	0.557
HbA1c	-0.005	0.918
Log HOMA-IR	0.168	<0.001
WC	0.131	0.006

adjusted for the following variables including BMI		

	β coefficient	P value

Gender	0.015	0.732
Age	0.111	0.018
Systolic BP	0.008	0.893
Diastolic BP	0.148	0.010
Triglycerides	0.050	0.280
HDL-c	0.019	0.678
HbA1c	0.013	0.768
Log HOMA-IR	0.147	0.003
BMI	0.181	<0.001

## 4. Discussion

All previous studies confirmed that abdominal fat distribution was significantly associated with insulin resistance. Chavez AO showed that simple morphometric measurements of adiposity/obesity, (i.e. abdominal circumference) could explain 59% of total insulin mediated glucose uptake, and provided a feasible method to screen and identify insulin resistant in baboons [16]. Miyazaki Y showed that visceral fat dominant accumulation as opposed to subcutaneous fat accumulation was associated with hepatic insulin resistance, independent of the individual's body type, in male patients with type 2 diabetes [17]. Recently a study showed that epicardial adipose tissue (EAT), as part of visceral fat, was also significantly increased in patients with metabolic syndrome and cardiovascular disease [18]. Moreover, lifestyle intervention involving diet combined with initial or delayed initiation of physical activity resulted in clinically significant weight loss and favorable changes in insulin resistance among patients with severe obesity [19].

How do we evaluate the role of low-grade systemic inflammation in the association between insulin resistance and obesity? Previous studies that examined the relationships among insulin resistance, CRP, and obesity were inconsistent. Several studies demonstrated that insulin resistance, as expressed by HOMA-IR, was significantly correlated with CRP concentrations in nondiabetic general populations. However, this correlation was abolished after adjusting for the parameters of obesity [7,8]. Similar observations were also made in a population that was at high risk for the development of type 2 diabetes mellitus [10]. The vigorous ongoing debate regarding the association among chronic inflammation condition, insulin resistance and obesity provided a conflicting hypothesis that chronic inflammatory state originates from obesity and drives the insulin resistant condition.

However, some studies concluded that the relationship between CRP concentrations and insulin resistance was independent of obesity. In the Insulin Resistance Atherosclerosis Study, Festa et al showed that CRP was strongly associated with insulin sensitivity, as assessed by a frequently sampled intravenous glucose tolerance test; and this association was independent of BMI [2]. Another study also demonstrated a strong association of fasting insulin with CRP concentration even after adjusting for BMI [5]. Gupta AK et al revealed the prediabetes group had significantly higher CRP and fibrinogen than those in normoglycemic group from healthy disease free obese adults in a weight loss study. This study suggested that a higher degree of systemic inflammation was associated with insulin resistance independent of obesity, concurrent prediabetes with prehypertension [20]. Chou HH et al concluded that insulin resistance was associated with C-reactive protein independent of abdominal obesity in nondiabetic Chinese population [6]. In this study we demonstrated that obesity and insulin resistance were strongly associated with CRP. These data extended previous reports done predominantly in nondiabetic subjects. To more precisely define the relationship between insulin resistance and CRP concentration, we further examined this relationship after adjusting for some metabolic factors in addition to age and gender. A positive association of HOMA-IR with CRP concentration was still evident after adjusting for waist circumference, indicating that this relationship was independent of abdominal obesity in the patients with type 2 diabetes. Our results were consistent with another Asian nondiabetic population study that demonstrated that the strength of the association between HOMA-IR and CRP was reduced, but the association remained statistically significant after adjusting for BMI [4,5]. Gallagher EJ considered that insulin resistance might be the underlying cause for the metabolic syndrome. Insulin resistance could be present in both obese and lean individuals, and was associated with inflammation. The mechanism that insulin resistance correlated with inflammation independent of central obesity was partly explained by this review [21].

Recently Nayak S et al revealed that adiponectin was not correlated with inflammatory marker [22]. And among non-obese persons, adiponectin correlated negatively with IL-6 and HOMA-IR [23]. So the debates on the association among inflammation state, insulin resistance and obesity may continue, and some further studies should be done.

Our study has several strengths. The subjects we included were all of population-based Han-Chinese patients diagnosed with type 2 diabetes. Besides, we used a uniform protocol, including standardized clinical assessments, anthropometric measurements, and biochemical measurements in a centralized core laboratory. However, some possible limitations of our study should be mentioned. First, because of the study's cross-sectional nature, our results do not establish causality. Second, subjects taking some antidiabetic and statin agents were not excluded from our study, which might have influenced the association of CRP levels and might have also affected the association of CRP with insulin resistance in the multivariable analysis.

In conclusion, our findings showed that insulin resistance was associated with CRP levels independent of abdominal obesity in Chinese patients with type 2 diabetes, suggesting that abdominal obesity could only partly explain the link between subclinical inflammation and insulin resistance.

## Competing interests

The authors declare that they have no competing interests.

## Authors' contributions

BL, YY and RH contributed to design of the study, analysis and interpretation of data, and drafting of the manuscript. ZY contributed to interpretation of data and critical revision of the manuscript. All authors have given the final approval of the version of the manuscript to be published.
